# Green synthesis of zinc oxide nanoparticles toward highly efficient photocatalysis and antibacterial application

**DOI:** 10.3762/bjnano.13.94

**Published:** 2022-10-07

**Authors:** Vo Thi Thu Nhu, Nguyen Duy Dat, Le-Minh Tam, Nguyen Hoang Phuong

**Affiliations:** 1 Faculty of Chemical & Food Technology, Ho Chi Minh City University of Technology and Education, 1 Vo Van Ngan, Thu Duc City, Ho Chi Minh City, Vietnam; 2 HUTECH University, 475A Dien Bien Phu Street, Binh Thanh District, Ho Chi Minh City, Vietnam

**Keywords:** green synthesis, methylene blue, methyl orange, rosin, ZnO nanoparticles

## Abstract

Zinc oxide nanoparticles (ZnO NPs) were successfully synthesized by a green method using rosin and zinc chloride as salt precursors. The phase structure, morphology, and particle size of ZnO were determined by X-ray powder diffraction, field emission scanning electron microscopy, and high-resolution transmission electron microscopy. The fabricated ZnO NP samples are crystalline with a grain size of 30–100 nm. The ZnO NPs were used as catalysts for the photodegradation of methylene blue (MB) and methyl orange (MO) under visible and UV light. The results indicate that the prepared ZnO material excellently removed MB and MO (*c*_initial_ = 10 mg/L) with efficiencies of 100% and 82.78%, respectively, after 210 min under UV radiation with a ZnO NP dose of 2 g/L. The photocatalyst activity of the synthesized material was also tested under visible light radiation with the same conditions; however, it achieved lower efficiencies. In addition, ZnO NPs were also tested regarding their antibacterial activity, and the results showed that the prepared ZnO samples had the highest (i.e., 100%) antibacterial efficiency against *E. coli*.

## Introduction

Currently, industrial development has generated a large number of pollutants which are released into the environment. The textile industry is one of the sources of organic pollution which is harmful to the environment and humans. Various technologies have been proposed to remove organic pollutants from water, including coagulation coupled with sedimentation, biological processes, membrane filtration, adsorption, advanced oxidation, catalysis, and photocatalysis [1–3]. Using semiconductors as photocatalysts has been a widely studied approach for the complete removal of organic pollutants due to their advantages. Semiconductors can act as catalysts for the complete degradation of organic substances when excited by light with an energy value higher than their bandgap. Among many semiconductors, TiO_2_ and ZnO are widely used as photocatalysts. ZnO has a higher quantum efficiency than that of TiO_2_ since it absorbs more energy in the UV region [4–7]. Furthermore, ZnO is a low-cost photocatalyst with high photocatalytic activity, nontoxicity, light sensitivity, and stability [8–10]. The photodegradation of organic substances by the use of ZnO catalysts occurs when ZnO is illuminated by light. When excited by light with an energy greater than the bandgap of ZnO, electrons from the valence band (VB) are excited to the conduction band (CB) to form photogenerated electrons in the CB and photogenerated holes in the VB [11–12]. These photogenerated electrons and holes migrate to the surface of ZnO to react with H_2_O and O_2_ to generate O_2_^•−^ and ^•^OH radicals, which oxidize organic substances. In addition, ZnO nanoparticles (NPs) have high antibacterial activity against bacteria, high biocompatibility, and are nontoxic to human cells [12–13]. Many studies have proven the antibacterial effect of ZnO [14–15] and also that nanoscale ZnO has a more effective antibacterial activity than that of large ZnO [12,16].

Various approaches for synthesizing nanosized materials have been investigated. Among these approaches, chemical and physical routes have their own disadvantages, such as adverse environmental effects due to the use of environmentally unfriendly chemicals or to the release of heat into the environment [17]. Therefore, the development of green approaches is necessary. The biosynthetic process using plant extracts as an alternative route is a promising method for synthesizing nanomaterials due to its rapid, low-cost protocol, and safety to the environment [18]. Numerous studies applied green methods for the synthesis of ZnO nanoparticles from plants, fruits, plant extracts, and seaweeds [19–22]. Rafaie et al. [23] used *Citrus aurantifolia* extracts to synthesize ZnO NPs with a size range of 50–200 nm. Sangeetha et al. and Gunalan et al. used *Aloe vera* leaves as a precursor to synthesize ZnO with a size range of 25–45 nm [24–25]. Many studies have synthesized nanosized ZnO for antibacterial and photocatalyst applications. Nava et al. [26] prepared ZnO NPs using *Camellia sinensis* extracts and applied ZnO NPs to degrade methylene blue (MB). Ambika et al. [12] synthesized ZnO by a green method using a precursor from the *Vitex negundo* plant extract and zinc nitrate, and antimicrobial properties of ZnO NPs were demonstrated against *E. coli* and *S. aureus* bacteria.

As mentioned previously, plant extracts were used as common precursors for nanomaterial synthesis due to their relatively high levels of the steroids, saponins, carbohydrates, and flavonoids which act as reducing agents and phytoconstituents as capping agents, providing stability to the nanoparticles [27]. Rosin is also a plant-derived material containing different resin acids, especially abietic acid and pimaric acid with a general chemical formula of C_20_H_30_O_2_ (C_19_H_29_COOH) [28]. It is the inexpensive and eco-friendly solid byproduct obtained after refining turpentine from *Pinus latteri* trees to produce turpentine oil, which is usually used as a precursor for many industrial applications such as paints, inks, adhesives, soap, and glue production [28]. Obviously, there is a high potential of using rosin as a green precursor for nanomaterial synthesis. Lack of information regarding this application has been reported. This is the motivation for this study which is focused on synthesizing nanosized ZnO materials by the sol–gel method using rosin as a green precursor. ZnO NPs were synthesized by a sol–gel two-step method from the green precursor rosin and zinc chloride salt. The antibacterial activity of the synthesized ZnO material against *Escherichia coli* (*E. coli*) was studied. In addition, the study also determined the ability of ZnO NPs to act as photocatalysts and to degrade dyes including MB and methyl orange (MO).

## Experimental Design

### Materials

Rosin was purchased from a Vietnamese company (Loc Thien Investment development Co., Ltd.), which was produced by distilling turpentine from *Pinus latteri* trees in Vietnam. The rosin used in this study is a clear, hard, brittle solid, which is light yellow to pink in color. Zinc chloride (ZnCl_2_) and sodium hydroxide (NaOH) were provided by Xilong Scientific Co., Ltd. Methylene blue and methyl orange were purchased from Merck Co., Ltd. Nutrients and agar powder were provided by Titan Co., Ltd. All reagents were of analytical grade. ZnCl_2_ and NaOH were diluted in DI water with low conductivity (0.06 µS/cm). *Escherichia coli* (*E. coli* VTCC-B-482) was used for the antibacterial experiments and purchased from the Hanoi National University.

### Preparation of zinc oxide nanoparticles by green syntheses

**Figure 1:**
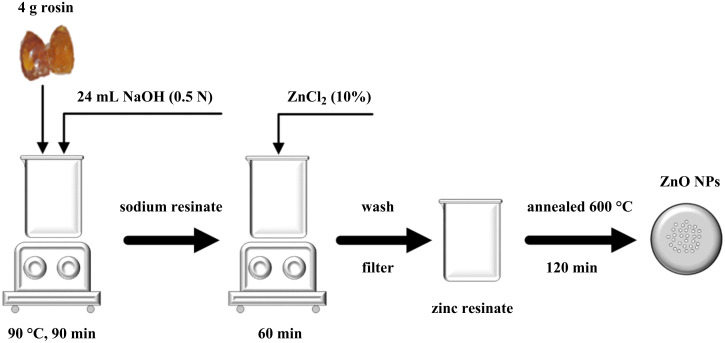
Schematic illustration of the synthesis of ZnO nanoparticles.

The ZnO NPs were synthesized by two steps. Firstly, the saponification reaction of rosin was performed using NaOH to create sodium resinate, and zinc resinate was subsequently synthesized from sodium resinate. Rosin (4 g) was dissolved in 24 mL of 0.5 N NaOH at 90 °C under magnetic stirring for 90 min. Then, 40 mL of ZnCl_2_ (10%) was added to the mixture which was stirred for 60 min to form zinc resinate. The suspension containing zinc resinate was then filtered and washed with hot distilled water to remove impurities. The zinc resinate was then calcined in a furnace with an increasing temperature rate of 5 °C/min to 600 °C and held for 30 min to obtain pure ZnO nanoparticle powder. The relevant reactions that form zinc resinate are shown in Equation 1 and Equation 2. The schematic illustration of the synthesis of ZnO nanoparticles is shown in Figure 1.


[1] $${\text{C}}_{19}{\text{H}}_{29}\text{COOH}+\text{NaOH}\to {\text{C}}_{19}{\text{H}}_{29}\text{COONa}+{\text{H}}_{2}\text{O}$$

[2] $$2{\text{C}}_{\text{19}}{\text{H}}_{\text{29}}\text{COONa}+{\text{ZnCl}}_{2}\to {\left({\text{C}}_{19}{\text{H}}_{29}\text{COO}\right)}_{2}\text{Zn}+2\text{NaCl}$$

### Methods for determining the characterization of the synthesized material

The phase of the synthesized material was determined by X-ray diffraction (XRD) using a Bruker D8 advanced X-ray diffractometer equipped with Cu Kα radiation (λ = 1.5418 Å). The morphology and size of the synthesized material were determined by field emission scanning electron microscopy (FESEM) on a Hitachi S-4800 at 15 kV and high-resolution transmission electron microscopy (HR-TEM) on a JEOL JEM-2100. The thermal decomposition of zinc resinate to form ZnO NPs were studied by thermal gravimetric analysis (TGA) and differential thermal analysis (DTA) curves using thermal gravimetric analysis (DSC131, LABSYS TG/DSC1600, TMv), by heating up to 1000 °C at a heating rate of 10 °C/min. The zeta potential was measured by analyzing 0.1 g of ZnO in 10 mL of water using a Malvern ZetasizerPro. The solid UV–vis DRS was carried out using a JASCO V550 UV–vis spectrometer.

### Photocatalytic degradation reaction

The photocatalytic degradation of a dye solution under visible and UV light using green-synthesized ZnO nanoparticles from rosin and zinc chloride salt was investigated using a batch photocatalytic reactor. Firstly, 0.1 g of ZnO NPs was added to 50 mL of MO or MB solution with an initial concentration of 10 mg/L. The solution was then submitted to magnetic stirring in the dark for 30 min to equilibrate the adsorption. Subsequently, the suspensions were illuminated by a 30 W LED or an 18 W high-pressure mercury lamp. The light source was fixed at a distance of 20 cm from the surface of the reactor. After fixed illumination times, 5 mL was withdrawn and centrifuged at 6000 rpm to measure the dye concentrations. The dye concentration was analyzed by UV–vis spectroscopy (Hitachi U2900) with maximum adsorption wavelengths of MB and MO of 664 nm and 464 nm, respectively [29–30]. The degradation efficiency was calculated using Equation 3.


[3] $$\text{degradation efficiency (\%)}=\frac{c_{0}-c_{\text{t}}}{c_{0}}\times 100\%,$$

where *c*_0_ is the initial dye concentration and *c*_t_ is the dye concentration at a given reaction time.

### Determination of the antibacterial efficiency against *E. coli*

The experiment was conducted using three different concentrations of ZnO NPs (1, 5, and 10 mg/mL) and two concentrations of *E. coli* (5·10^4^ and 5·10^5^ CFU/mL). After adding ZnO NPs into the solutions containing *E. coli*, the suspensions were stirred. Samples were taken out at different contact times of 1, 3, and 6 h to evaluate the influence of time on the antibacterial efficiency. The solution was obtained after being diluted to decimal concentrations of 1/10, 1/100, and so forth. After that, 100 μL of the solution containing *E. coli* was taken using a micropipette and spread on a plate containing the *E. coli* culture medium (consisting of 2.6 g of nutrient broth and 2 g of agar in 200 mL of distilled water). The plate was incubated at 37 °C for 24 h to determine the amount of *E. coli* that survived (i.e., by counting colonies). Each experiment was repeated three times. The *E. coli* inhibition percentage was calculated via Equation 4:


[4] $$E.\;coli\text{ inhibition percentage }(\%)=\frac{c_{0}-c_{\text{i}}}{c_{0}}\times 100\%,$$

where *c*_0_ is the initial number of *E. coli* cells and *c*_i_ is the average number of *E. coli* cells/plate.

## Results and Discussion

### Characteristics of synthesized ZnO NPs

**Figure 2:**
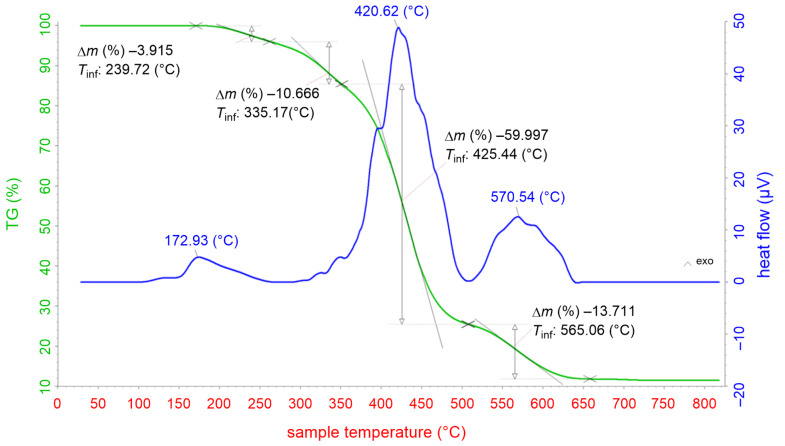
DTA/TG diagram of the zinc resinate sample.

Figure 2 shows the results of DTA/TG analysis of zinc resinate. The TGA curve at a temperature range between 30–300 °C shows an exothermic peak at 172.93 °C and a weight loss of 7%, which corresponds to dehydration of physically adsorbed water. At temperatures between 300–510 °C on the TG curve, a mass decrease of 61% was observed, whereas on the DTA curve, the exothermic peak at 420.62 °C corresponds to the decomposition of an organic compound. Finally, at temperatures in the range of 520–800 °C, the weight loss was found to be 32%. The DTA curve indicated an exothermic peak at 570.54 °C, revealing that the decomposition of organic compounds continues and the oxidation of Zn to form the ZnO crystalline phase occurs completely. From there, the organic matter in the zinc resinate begins to decompose at 300 °C. At a temperature of about 600 °C, the organic matter is almost completely decomposed and only the zinc metal is oxidized to form zinc oxide.

**Figure 3:**
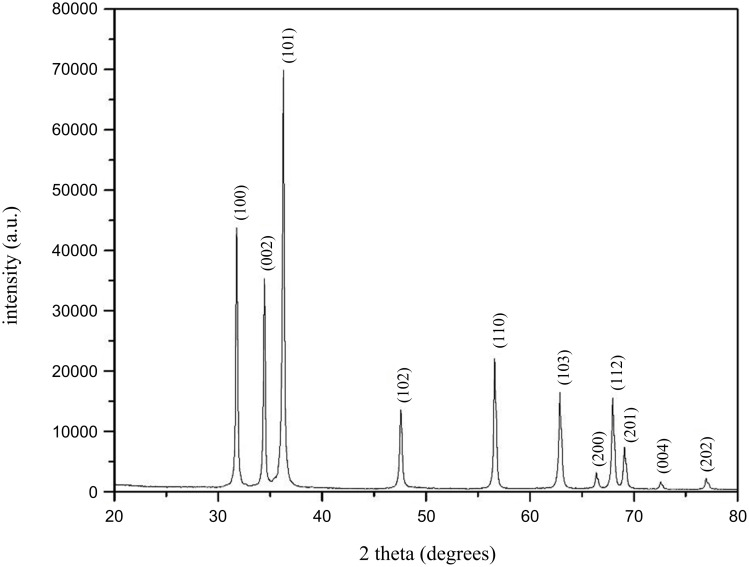
XRD diagram of ZnO NPs.

The XRD diagram of ZnO samples (Figure 3) shows diffraction peaks located at 2θ = 31.81°; 34.44°, 36.26°, 47.57°, 56.64°, 62.88°, 66.41°, 67.96°, 69.12°, 72.72°, 77.01° corresponding to the crystal planes (100), (002), (101), (102), (110), (103), (200), (112), (201), (004), (202) which characterize the ZnO wurtzite hexagonal structure (JCPDS card no 36-1451). In addition, there are no characteristic peaks of other crystals. The XRD results are consistent with the results of the DTA mentioned above. Sodium resinate was formed from rosin and sodium hydroxide as shown in Equation 1. Then, the exchange reaction between sodium resinate and zinc salt to produce zinc resinate was conducted as shown in Equation 2. The obtained zinc resinate is heated at 600 °C to be completely decomposed and zinc is oxidized by oxygen to form the ZnO crystalline phase. The ZnO crystal size presented in Table 1 (14–22 nm) was obtained from the XRD spectrum using the Debye–Scherer formula (i.e., Equation 5):


[5] $$L=\frac{k\lambda }{\beta \cos\theta },$$

where *L* is the ZnO crystal size (nm), *k* = 0.9, λ = 0.15418 nm [30], θ is the Bragg angle, and β is the full width at half maximum (FWHM).

**Table 1:** Crystal size of ZnO determined from the Debye–Scherer formula.

Crystal plane	2θ	FWHM (b)	B (rad)	*L* (nm)

(100)	31.81	0.437	0.0076	21.38
(002)	34.44	0.385	0.0067	21.60
(101)	36.26	0.437	0.0076	19.13
(102)	47.57	0.613	0.0107	14.16
(110)	56.64	0.642	0.0112	14.06
(103)	62.88	0.689	0.0120	13.51
(112)	67.96	0.611	0.0110	15.68
(201)	69.12	0.556	0.0097	17.35

**Figure 4:**
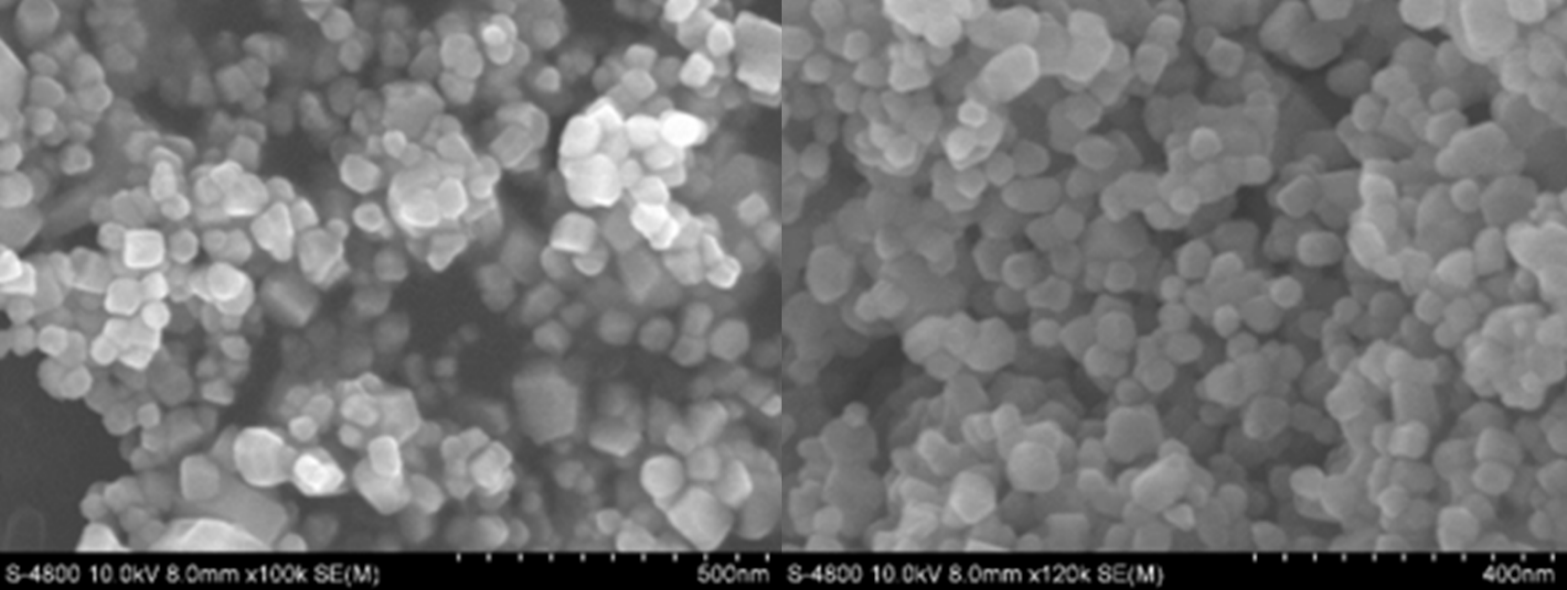
FESEM image of synthesized ZnO NPs.

**Figure 5:**
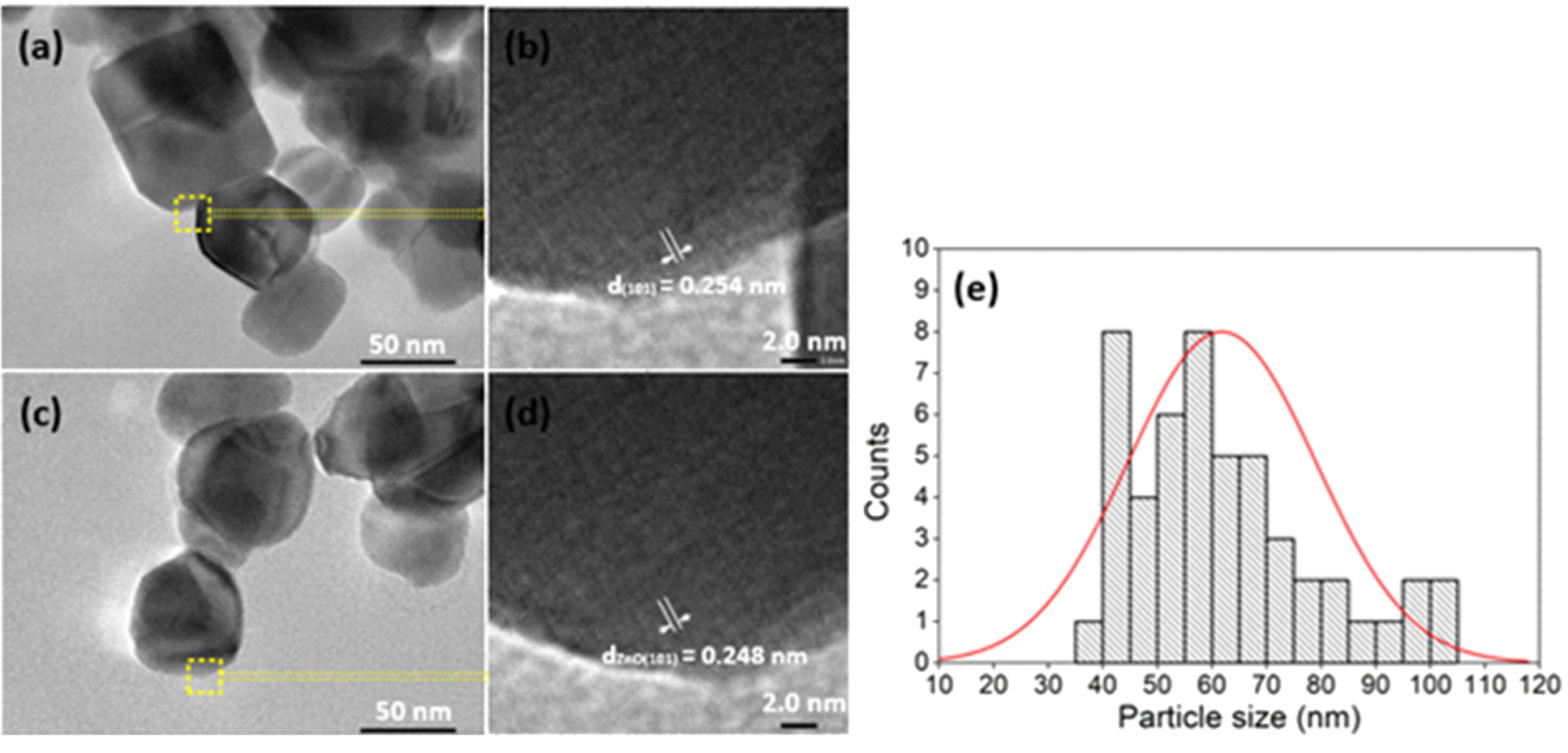
HR-TEM images of synthesized ZnO NPs.

The morphology and size of ZnO NPs were illustrated using FESEM and HRTEM. The FESEM image shown in Figure 4 indicates that ZnO NPs have a relatively homogeneous size. The HR-TEM results and particle size distributions obtained from the HR-TEM images are shown in Figure 5. The HR-TEM images in Figure 5a and Figure 5b show the interplanar spacing of 0.251 ± 0.003 nm corresponding to the (101) crystal plane of ZnO. The size of ZnO NPs is in the range of 30–100 nm (Figure 5c and Figure 5d) and the average size of ZnO NPs from the distribution graph (Figure 5e) was about 60 nm in diameter. Furthermore, the results are in line with those reported by other studies. Stan et al. [31] used plant extracts (garlic, onion, and parsley) to synthesize ZnO NPs and made ZnO NPs of 20–70 nm in size. Hassan et al. [32] synthesized ZnO using *Coriandrum sativum* leaf extract and zinc acetate dihydrate and synthesized ZnO NPs with sizes in the range of 9–18 nm.

**Figure 6:**
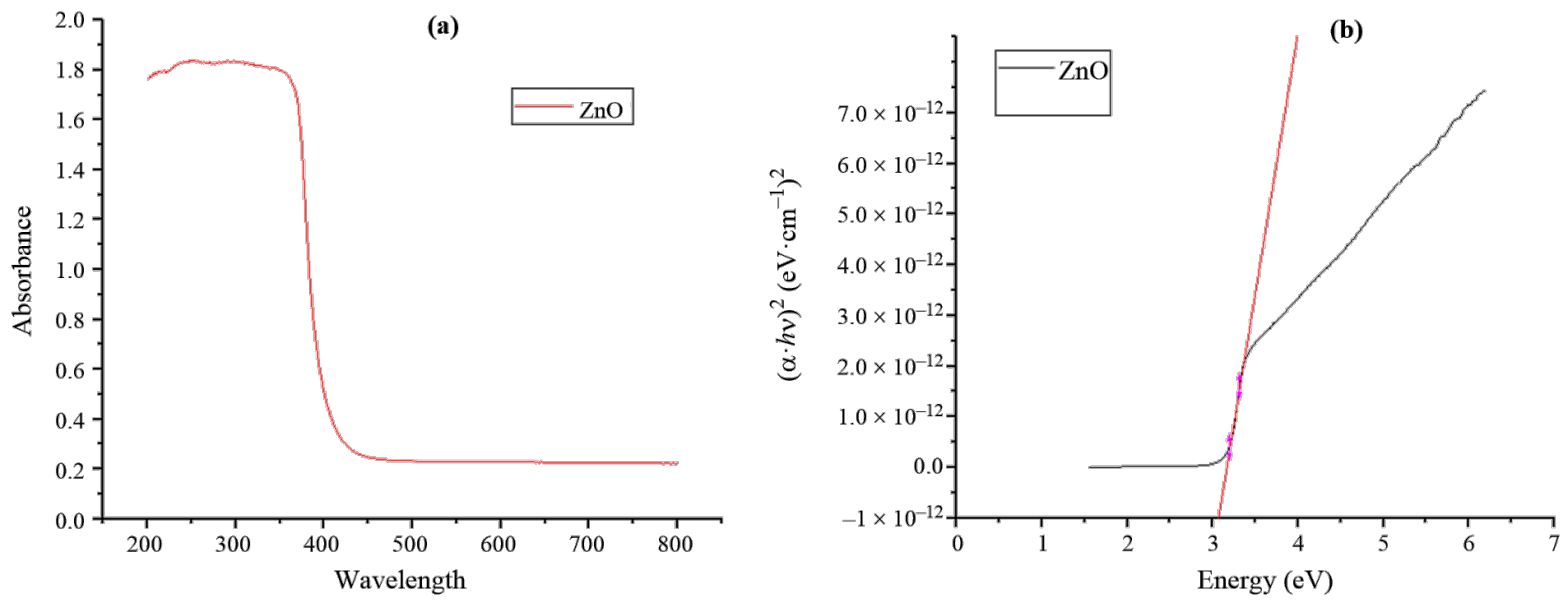
UV–vis DRS spectra (a) and plot of (α·*h*ν)^2^ as a function of photon energy for ZnO NPs (b).

**Figure 7:**
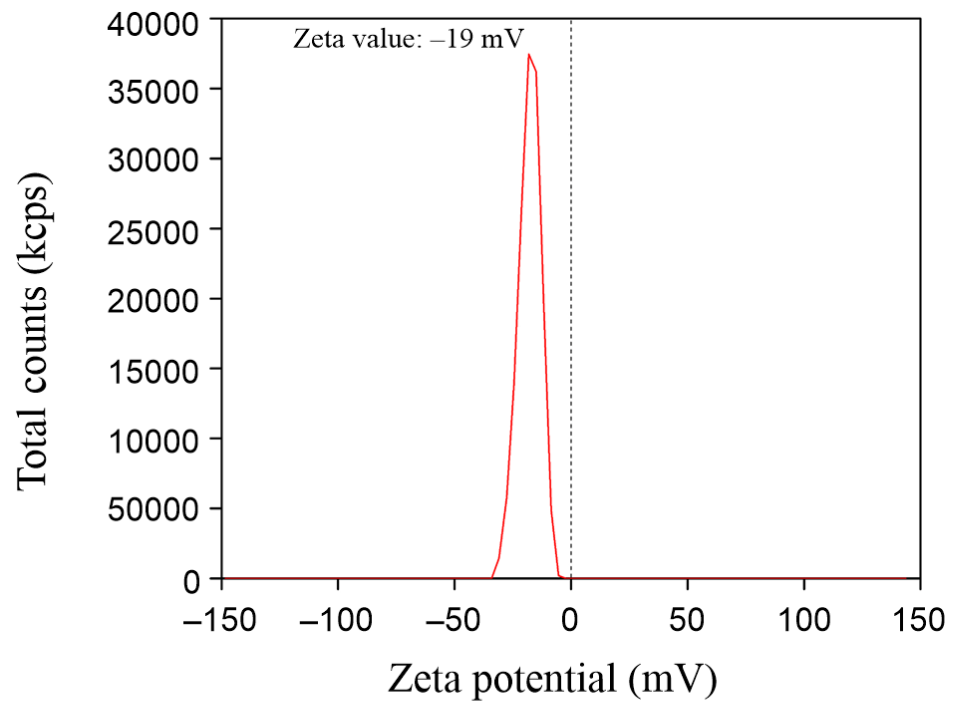
Zeta potential of synthesized ZnO NPs.

UV–vis DRS spectra of ZnO were shown in Figure 6a. ZnO absorbs light in the ultraviolet region. The bandgap energy of synthesized ZnO was determined by extrapolation of the linear part of the curve (α·*h*ν)^2^ as a function of photon energy (Figure 6b). The bandgap energy of synthesized ZnO was 3.15 eV, which is close to the bandgap value of 3.2 eV of ZnO shown in a previous report [33]. The zeta potential value displays the surface charge and stability of ZnO NPs. In this study, the zeta potential of ZnO NPs was −19 mV (Figure 7), indicating that ZnO NPs are negatively charged and stable in aqueous solution.

### Photocatalytic degradation of dyes

**Figure 8:**
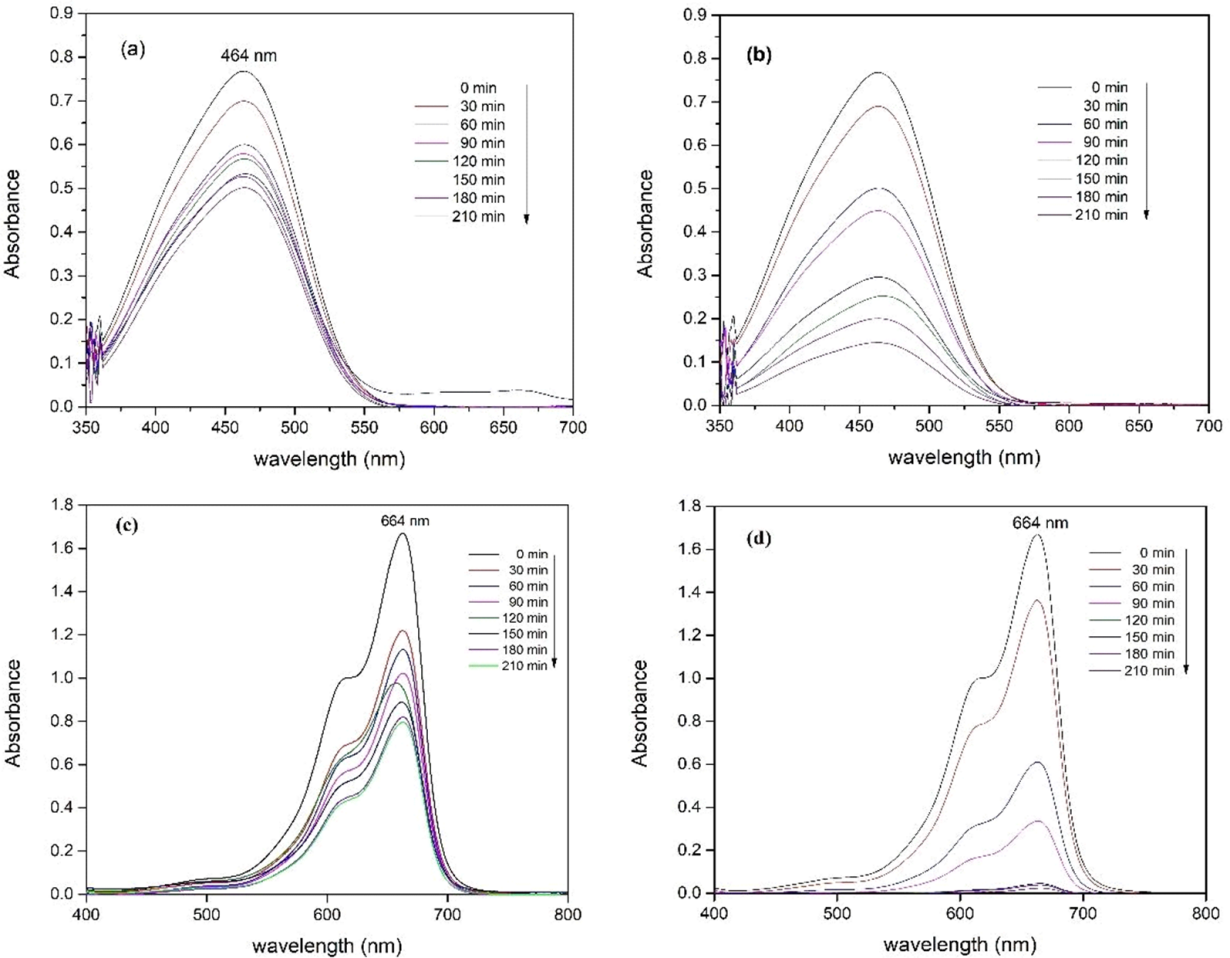
UV–vis spectra of degradation of MO (a, b) and MB (c, d) under visible and UV light.

**Figure 9:**
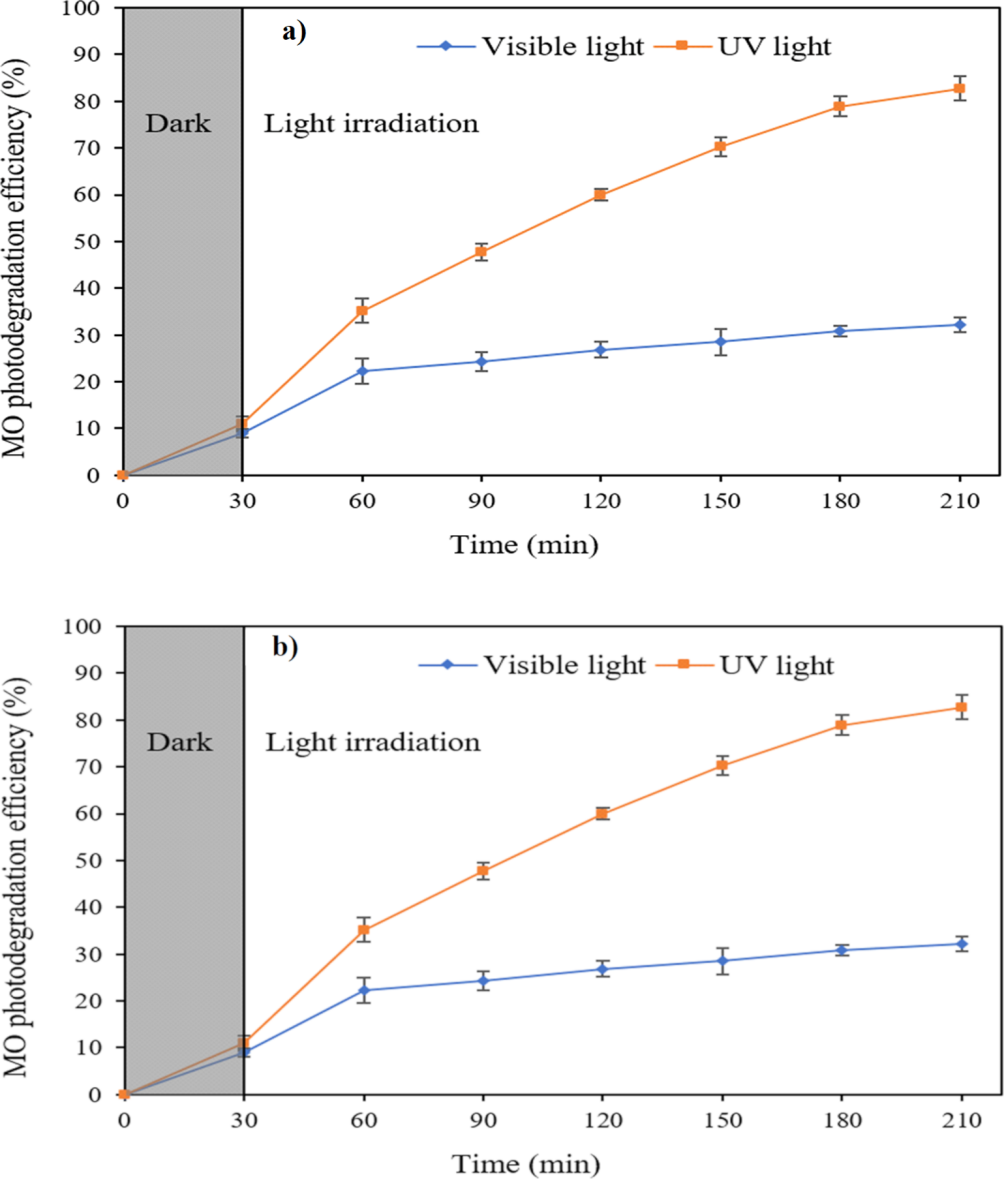
Photodegradation efficiency of MO (a) and MB (b) under visible and UV light.

The photocatalytic degradation of ZnO NPs was evaluated through the degradation of methylene blue and methyl orange under visible and UV light and the degradation efficiency was calculated via Equation 3. Figure 8 shows the degradation absorption spectra of MO and MB by synthesized ZnO under visible and UV light for different time intervals. The intensity of the peak decreased with increasing irradiation time. The results in Figure 9 show that the degradation efficiency of MB and MO solutions under UV light was higher than that under visible light. The degradation of MB (10 mg/L) under visible and UV light by ZnO NPs was 50.46% and 100%, respectively, after 210 min of irradiation. The degradation efficiency of MO (10 mg/L) by ZnO was lower than that of MB (10 mg/L). After 210 min of irradiation by visible and UV light, the MO degradation efficiency by ZnO NPs reached 33.56 and 82.78%, respectively. ZnO has a rather high bandgap energy; therefore, the degradation efficiency of organic substances under visible light is not as high as that under UV light. Maddu et al. [34] studied the preparation of ZnO NPs with a size of 30 nm. The degradation efficiency of MB (5 mg/L) after 150 min of irradiation by visible and UV light was 40 and 80%, respectively, while synthesized ZnO NPs in this study can degrade more than 90% of MB (10 mg/L) under UV light after 150 min.

### Antibacterial performance of synthesized ZnO NPs

**Figure 10:**
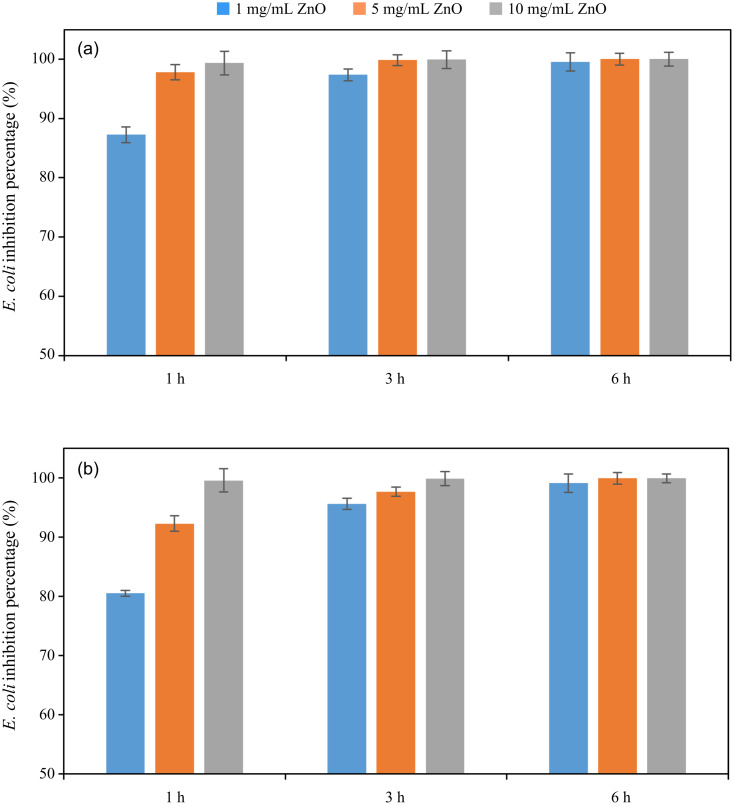
*E. coli* inhibition percentage by ZnO with different amounts of ZnO in various contact time intervals, with *E. Coli* initial concentrations of 5·10^4^ CFU/mL (a) and 5·10^5^ CFU/mL (b).

The experimental results on antibacterial performance of synthesized materials are presented in Table 2, the *E. coli* inhibition percentage was calculated via Equation 4. Figure 10a shows the results of inhibition efficiency of *E. coli* at an initial concentration of 5·10^4^ CFU/mL by ZnO NPs with ZnO NPs doses of 1, 5, and 10 mg/mL and contact time intervals of 1, 3, and 6 h. The ZnO NP concentration of 1 mg/mL is not enough to inhibit all *E. coli* bacteria after 6 h of treatment. By increasing the concentration of ZnO NPs to 5 mg/mL at contact times of 3 and 6 h, the inhibitory efficiency reached 99.83 and 100%, respectively. When the dose of the photocatalyst increased to 10 mg/mL, the *E. coli* inhibition efficiency reached 99.35% with a contact time of 1 h and the efficiency was 100% when the contact time was 3 h. The results of *E. coli* bacteria inhibition by ZnO NPs synthesized by the green method when the initial concentration of *E. coli* is increased to 5·10^5^ were shown in Figure 10b. When the concentration of ZnO was 1, 5, and 10 mg/mL, the inhibitory efficiency on *E. coli* for 1 h was lower than that on *E. coli* at a concentration of 5·10^4^ CFU/mL. When increasing the ZnO NP concentration to 5 mg/mL, the *E. coli* inhibition efficiency reached 99.96% in 6 h and the *E. coli* inhibition efficiency reached 100% when the ZnO NPs dose was increased to 10 mg/mL with a contact time of 6 h.

**Table 2:** Photographs of *E. coli* bacterial culture plates formed after *E. coli* plates were exposed to ZnO NPs at different times.

Content of ZnO NPs	*E. coli* initial concentration: 5·10^5^ CFU/mL			*E. coli* initial concentration: 5·10^4^ CFU/mL		
	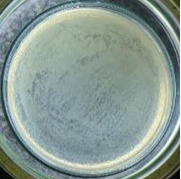			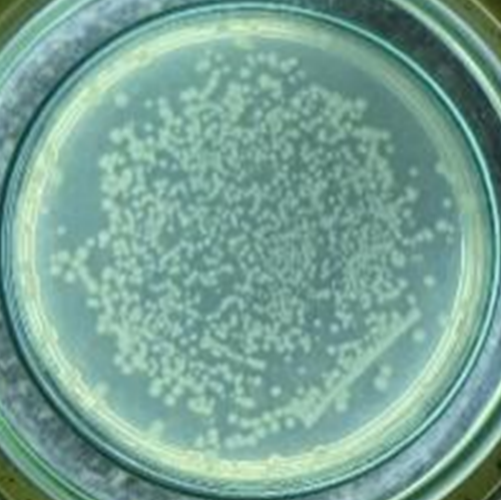		

	1 h	3 h	6 h	1 h	3 h	6 h

10 mg/mL ZnO	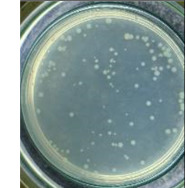	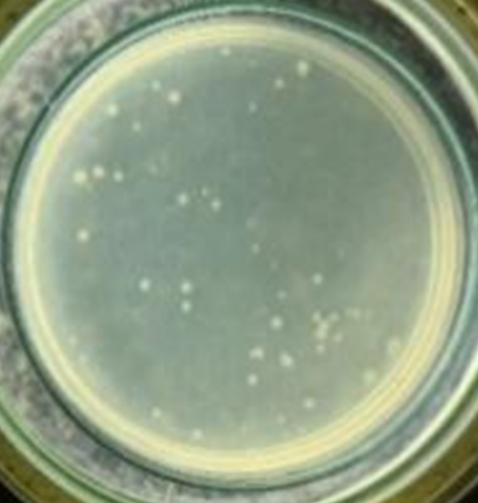	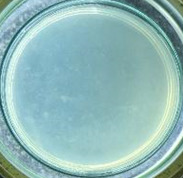	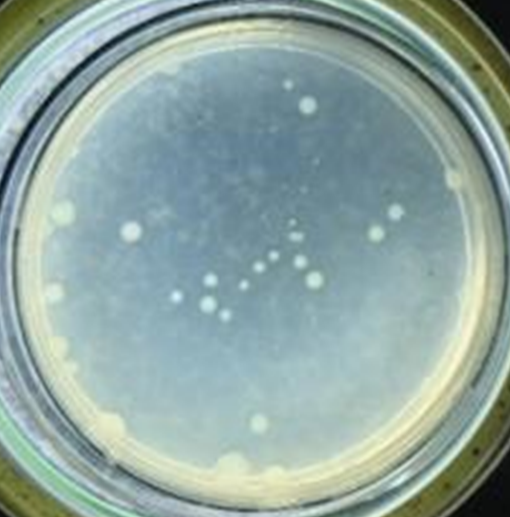	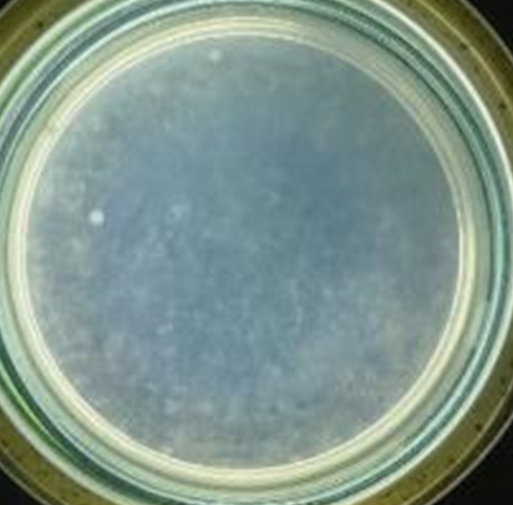	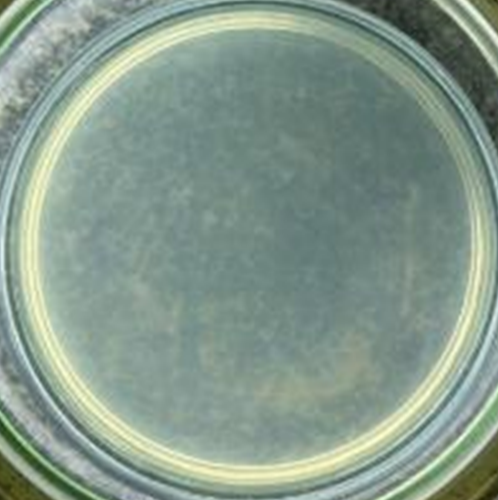
5 mg/mL ZnO	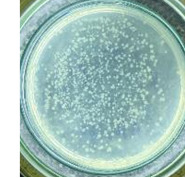	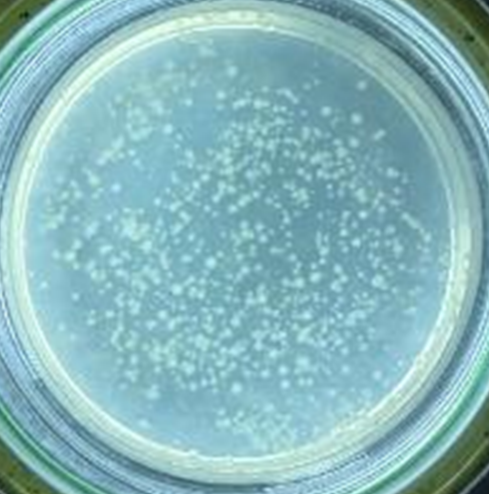	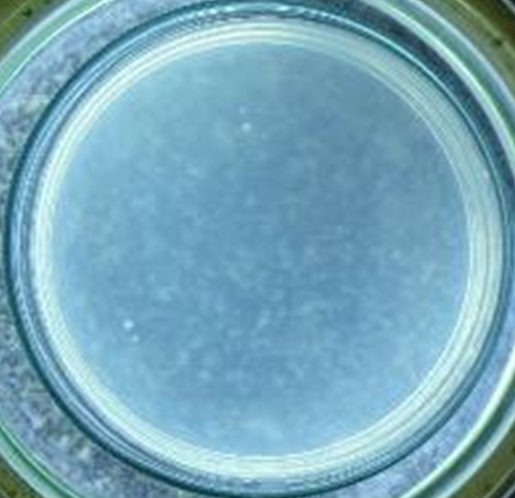	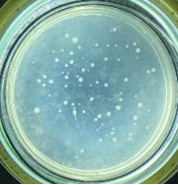	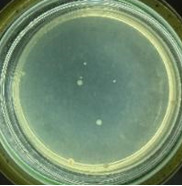	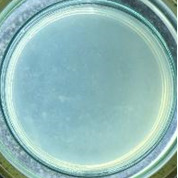
1 mg/mL ZnO	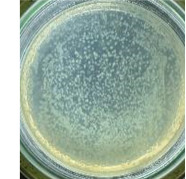	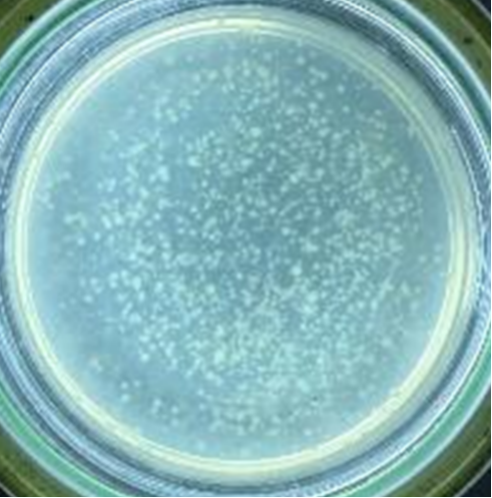	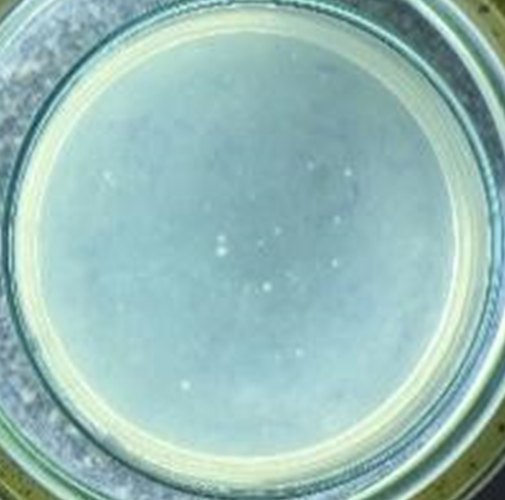	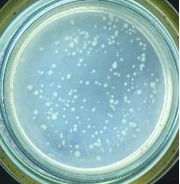	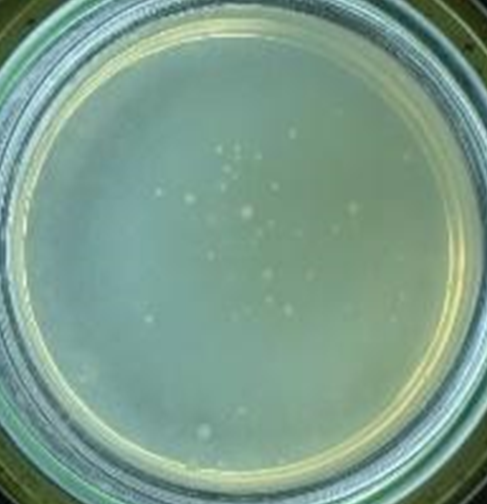	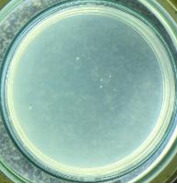

### Proposed mechanism of photocatalytic dye degradation and antibacterial activity against *E. coli* by ZnO NPs

**Figure 11:**
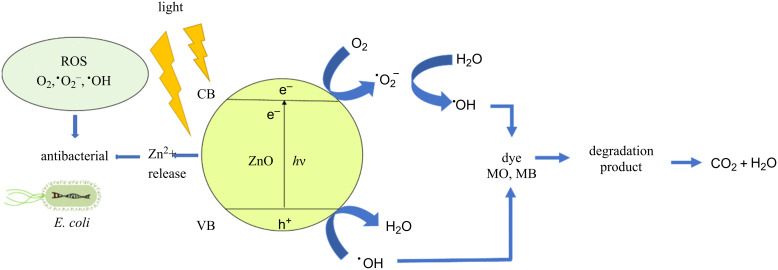
Proposed mechanism of photocatalytic dye degradation and antibacterial activity against *E. Coli* by synthesized ZnO NPs.


The mechanism of photocatalytic degradation and *E. coli* antibacterial activity by ZnO NPs is illustrated in Figure 11. When ZnO is irradiated with visible or UV light whose energy is equal or greater than the bandgap of ZnO, the electrons from the VB of ZnO NPs are excited to the CB generating holes in VB and electrons in CB. The electrons reduce the oxygen absorbed on the ZnO surface to form ^•^O_2_^−^. These ^•^O_2_^−^ species continue reacting with H_2_O to form H_2_O_2_ and ^•^OH. The hole in VB will react with H_2_O to produce ^•^OH radicals. These ^•^OH radicals are strong oxidizing radicals and are mainly present in solution which can degraded dyes. These radicals can attract MO and MB molecules, oxidize the dye molecules to degradation products, and finally completely degrade the dyes to CO_2_ and H_2_O [35–36].

[6] H2O+ZnO (hVB+)→ZnO+O•H

[7] O2+Zn (eCB−)→O•2−

[8] O•2−+H2O→HO2•+OH−

[9] $${\text{HO}}_{2}{}^{•}+{\text{HO}}_{2}{}^{•}\to {\text{H}}_{2}{\text{O}}_{2}+{\text{O}}_{2}$$

[10] O•2−+H2O2→HO−+O2+O•H

In addition, when ZnO NPs get in contact with *E. coli*, reactive oxygen species (ROS), such as ^•^OH, ^•^O_2_^−^, O_2_ formed on the surface of ZnO NPs, will break bacterial cell membranes and enter the cells destroying organelles and ultimately inhibiting and shutting off bacteria metabolism [37–38]. The ^•^OH radical is the most active oxidant which rapidly reacts with bacterial nucleic acids, lipids, proteins, DNA, and amino acids [39–41]. The O_2_ causes biomembrane oxidation reactions, damaging tissues [42]. Furthermore, it is reasonable to explain that the additional toxicity that causes bacterial death is due to the fact that zinc solubilization releases Zn^2+^ ions which can infiltrate into bacterial cell membranes, inhibiting amino acid metabolism and disrupting the bacterial cell enzymatic system [43–45].

## Conclusion

ZnO nanoparticles were synthesized by a green method using rosin extracted from *Pinus latteri* trees in Vietnam. The XRD diagram of synthesized ZnO showed that ZnO has the hexagonal wurtzite structure form. The HR-TEM image showed the interplanar spacing of 0.251 ± 0.003 nm corresponding to the (101) crystal plane of ZnO, and the size of ZnO NPs is in the range of 30–100 nm. The bandgap energy of synthesized ZnO was 3.15 eV. Synthesized ZnO NPs showed high photocatalytic activity in dye degradation (methylene blue and methyl orange). The photodegradation efficiency of a dye solution by ZnO NPs under UV is greater than that under visible light. ZnO NPs completely degraded a MB solution of 10 mg/L for 210 min under ultraviolet light while the MB degradation efficiency achieved 50.46% under visible light. After 210 min of irradiation, the degradation efficiency values of a MO solution of 10 mg/L under visible and UV light by ZnO NPs were 33.56% and 82.78%, respectively. Furthermore, the synthesized ZnO NPs exhibited high efficiency against *E. coli* Gram-negative bacteria. The *E. coli* bacteria with concentrations of 10^5^ and 10^4^ CFU/mL was almost completely destroyed after 6 h by ZnO with concentrations of 1, 5, and 10 mg/mL.
